# Streamlining electronic reporting of serious adverse events (SAEs) using the REDCap data collection system: the eSAE Project

**DOI:** 10.1186/s13063-024-08317-0

**Published:** 2024-07-24

**Authors:** Joanna Black, Patrick Julier, Lucy Eldridge, Vicki S. Barber

**Affiliations:** Oxford Clinical Trials Research Unit (OCTRU), Nuffield Department of Orthopaedics, Rheumatology & Musculoskeletal Sciences (NDORMS), University of Oxford, Oxford, OX3 7LF UK

**Keywords:** SAE collection, SAE processing, SAE reporting, SUSAR, REDCap, Regulatory compliance

## Abstract

**Background:**

It is essential that electronic data collection (EDC) systems are both compliant with regulations and the principles of Good Clinical Practice (GCP) to allow for the timely and accurate reporting of data including safety data. For clinical trials of investigational medicinal products (CTIMPs), investigators must immediately report to the sponsor any serious adverse event (SAE) that occurs in a site for which they are responsible. It is therefore expected that sponsors provide systems for timely review and reporting should a SAE be classified as a suspected unexpected serious adverse reaction (SUSAR). Challenges arise when data related to adverse events (AEs) needs to be re-entered for SAEs; this can be prone to error and may delay reporting. Additionally, recognising what has changed from an initial SAE report when an investigator responds to queries raised can cause errors.

**Method:**

A multi-disciplinary working group came together from a UK academic clinical trials unit (CTU) to establish if an electronic system could be created in the unit’s open-source EDC system—REDCap, to manage SAEs in an efficient way.

**Results:**

A module has been created in REDCap to facilitate electronic SAE reporting: enabling an AE form to automatically trigger an SAE form for any AE which is also a SAE, prepopulating relevant fields of the SAE form, reducing the risk of delay and error when entering data into the SAE form. The system has also been developed with an embedded code to allow for instant visual recognition of any data updated following reporting to allow the sponsor to immediately review and resolve SAEs in a timely manner, complying with UK regulatory reporting. This functionality ‘The eSAE Project’ is now an active project for all of our new trials where data collection is undertaken using the REDCap system.

**Conclusion:**

The eSAE Project coded into REDCap offers a unique way of populating SAE forms with information already entered in the initial AE forms as applicable, coupled with highlighting any updates during the lifetime of the SAE for sponsors to identify any new information that needs to be reassessed to process and report the SAE.

## Background

The use of electronic data capture (EDC) systems in clinical trials to replace paper data collection has been increasing over the last 20 + years becoming the ‘preferred technology’ for clinical trials [1–3] leading to a wide variety of solutions being produced including commercial offerings, free/open source (e.g. Research Electronic Data Capture (REDCap)) software [4–7], and bespoke (i.e. custom) system creation [8]. The use of EDC systems and other digital data collection systems will only continue to increase over the next decade with funders wanting continued efficiency, especially with initiatives such as those reported by Inan et al. [9] to completely digitise clinical trials. This is also true for reporting of safety by site investigators, which until recently had the preferred method of reporting being the completion of a paper SAE form which was then submitted to the sponsor using a scanned copy of the form to either a dedicated secure email account, secure fax machine or via the post.

Whichever EDC system is used, it must be compliant with the principles of Good Clinical Practice (GCP) and the requirements set out in ICH E6, and the latest draft of this (R3) continues the importance of the data collection and the systems utilised to undertake this [10]. In addition, as per the EMA guideline on the use of computerised systems and electronic data in clinical trials [11]— any risks related to the use of computerised systems should be identified, analysed, and ideally mitigated, or justifications given where risks have to be accepted.

Of the EDC systems available to trialists, one that has gathered momentum in the academic trials space around the world is REDCap [4, 5]. This system has grown over time to provide increased functionality in areas such as eConsent [12], integration with interoperability standards [13] and mobile data collection [14, 15].

It is an UK regulatory requirement for SAEs that occur in a trial participant and identified in the protocol as needing immediate reporting to be reported within 24 h of the investigator’s knowledge of the event. With the advance of EDCs, it should now be possible for the sponsor to be in receipt of SAE information as soon as the investigator enters the information in the reporting tool. Such reporting tools can be validated to send email notifications to those concerned with the review and processing of the SAE. Providing the SAE contains the minimum criteria, on entry in the EDC, the regulatory clock ‘day 0’ kicks in and hence the review process must begin.

When the sponsor representative reviews the SAE data and sends queries to the investigator, it must be such that the site responds in a timely manner and any data that is updated or added in as newly completed fields is visibly obvious to the sponsor when the SAE is resubmitted to avoid any oversight. This ensures that the reviewer takes into account all new information provided to then proceed with the processing of the SAE and the conclusion of whether an SAE is a suspected adverse reaction (SAR) or suspected unexpected serious adverse reaction (SUSAR), in which case there will need to be the regulatory reporting to the appropriate bodies such as the MHRA for CTIMPs undertaken in the UK.

The clinical trial community in academia has struggled to find solutions for the remote reporting of SAEs which are not only cost-effective but which allow a streamlined way of collecting and processing the data with the input of the investigator being done in the timely manner.

Our clinical trials unit (CTU) formed a small multi-disciplinary working group composed of the Head of Programming, Head of Regulatory Affairs and Quality Assurance, Director of Clinical Trial Operations, and Senior Database Officer. The group was based on previous other groups working on different EDC systems to identify a solution which would improve the quality and speed of SAE collection and processing, and ensure that the SAE data being reviewed could be as accurate and up to date as is reasonably practicable, and any updates clearly flagged within the REDCap EDC system customisable functionalities [16]. For this project to be a success, it was important that different expertise was engaged and this included members from quality assurance, programming, database design and trial operational teams. In addition, any solutions to the above needed to be configurable to meet the varying requirements of different studies that are conducted within our CTU. This paper describes the system set up in REDCap to address the above within a test instance of the CTUs REDCap environment.

## Methods

A functionality specification was devised that needed to be met for any effective eSAE reporting system to be developed. Table 1 lists the minimal requirements identified by the team.

**Table 1:** Functionality specification for an efficient eSAE system

• Automated numbering of a new AE/SAE report
• Prepopulating duplicated fields in an SAE form from those in an AE form, or fields from other forms (e.g. treatment allocation, randomisation date from the randomisation form)
• Ability to flag initiation of a SAE report
• Ability to indicate SAE is ready for review and email to be sent to sponsor representative when SAE is ready to be reviewed
• Ability to update an SAE report
• Ability to flag any updates made by a site to an SAE report and email to be sent to sponsor representative to inform of update that needs to be reviewed
• Clear highlighting of any changes made to a report
• Ability for users of different types to interact/view/edit with the form according to role (CI, site, sponsor)
• Ability to see the history of all changes made

A REDCap development database was created with multiple different user roles (chief investigator, trial manager, site user) to allow full testing and replication of how an eSAE could be collected, reported, reviewed, followed up, and concluded. Template eAE and eSAE forms were designed and tested extensively to ensure that they collected sufficient information to meet regulatory requirements. The out-of-the-box REDCap functionality that was available was reviewed, and the CTU IT team developed additional modules to enhance the core functionality where needed to address the issues identified regarding eSAE reporting and processing. The testing that took place followed the standard practice of using test scripts based on the requirements in the User Requirement Specification (URS) to ensure that the code worked as per the requirements. During the development phase, there were demonstrations with several of our trial teams and their feedback further refined the system.

An ‘Auto Record Generation (eSAE)’ module was developed to auto-create an eSAE form when an eAE was completed and the user indicated that the event is an SAE after selecting the criteria that defines an AE as serious. The newly created eSAE was designed to be prepopulated with data from the eAE form, removing any possibility of transcription errors, and it was designed to be able to additionally pull in relevant data from other forms, such as a treatment allocation or medication type if that expanded functionality was required. The module was created to allow the amount and type of data to be prepopulated to be able to be configured differently for each study.

A bespoke ‘Label Generator’ module was also created to insert an AE log number (the format of which can be defined on a per-trial basis), counting either within sites or across the entire trial, which can be included in any auto-generated eSAE. This ensures sequential log numbers for efficient reporting and monitoring.

To address the need to restrict eSAE edit access to different sections by user role, an existing module was extended with tags to be applied at the field level; these allow fields to be hidden or made read-only to one or more user roles. These were added to an eSAE template form such that all site-entered data was made read-only to the sponsor and chief investigator, and the trial-office-use only sections (including reviewer assessments and MedDRA coding) were entirely hidden from site staff. The inbuilt REDCap alerts system was configured to instantly send an automated email to the trial inbox when certain criteria were met (e.g. the site staff section of the eSAE form has been filled out) with a link to review the form; this eliminated any potential delays in notification. Once the email alert is received in the trial inbox, this prompts the CTU to then log in the REDCap and review the SAE. The alert was then set to be re-triggered every time the eSAE form was updated, enabling the trial team to prompt the chief investigator to re-assess the data as necessary. The re-assessments were then aided by an internally developed ‘Highlight Changes’ module which made updated data points visually obvious by surrounding them with a bright border; a trigger for this highlighting and the border colouring can be defined and configured per study. In our module, we opted for red which is highly visible and also visible as brown/yellow for those who are colour blind.

The system was extensively tested against the requirement specification and using different roles and permissions across a number of test studies built in REDCap by both the project team and further members from the CTU. All feedback received through the test scripts were acted upon to ensure user acceptance testing had passed satisfactorily.

## Results

**Fig. 1:**
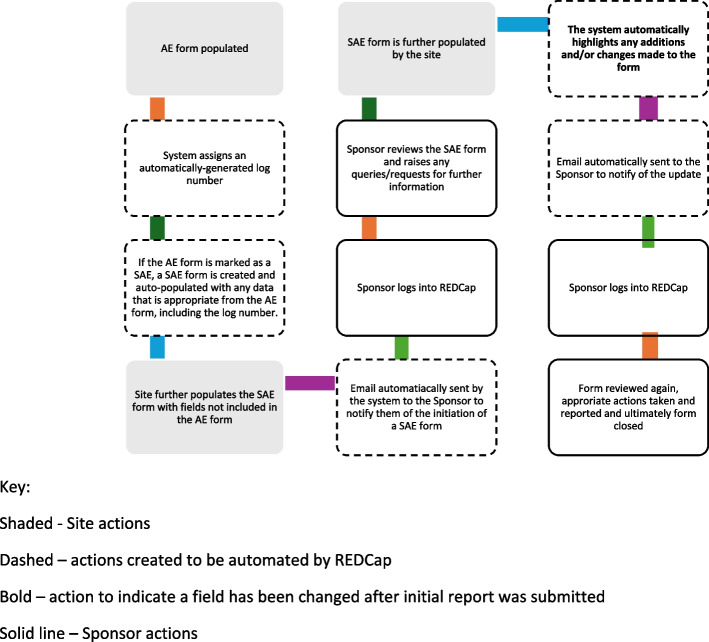
Overview of the eSAE Project/system created

Figure 1 shows a flowchart overview of the system produced to enable the eAE/SAE process that was developed using the requirements from the specification listed in the ‘Methods’ section above. The flowchart shows the processes which are led by the site, the automated functionality of the system that streamlines the process and those activities undertaken by the sponsor.

The system was designed such that if an AE qualifies as an SAE, once the AE has been automatically given an ID log number and the CRF is saved in REDCap a corresponding SAE form is automatically started and corresponding fields prepopulated with data from the AE, including the AE log ID. The user completing the AE form is presented with a message: ‘*As this AE also qualifies as a SAE, once you have saved this CRF, a corresponding SAE form will have been automatically started which you need to access to provide full details necessary for SAE reporting.*’ Users were then expected to further complete the initial partially populated SAE form providing as much detail as possible. The system then sent an automatic email notification to the sponsor representative to make them immediately aware that there is a SAE in the REDCap database ready to be reviewed and processed.

**Fig. 2:**
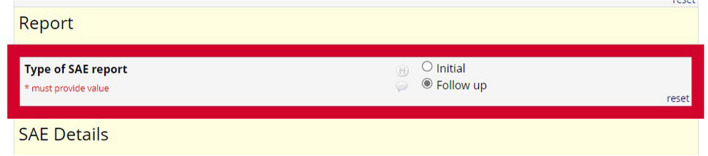
A highlighted field indicating change in information from initial report

After the initial submission, any changes made to the eSAE form by the investigator will automatically be highlighted by a red (or colour of choice) border around the field that has been changed. This will occur whether information has been updated or added in as new data in a field that was previously left empty. In this example (Fig. 2), the investigator has unticked ‘Initial’ and ticked ‘Follow up’, which instigated the appearance of the red highlight surrounding the field. An additional module added to our REDCap instance also prompts the user to provide a ‘reason for change’ every time changes are made to the fields in a form; all changes with a reason for change can be viewed on a report pulled through the history tab built within REDCap.

**Fig. 3:**
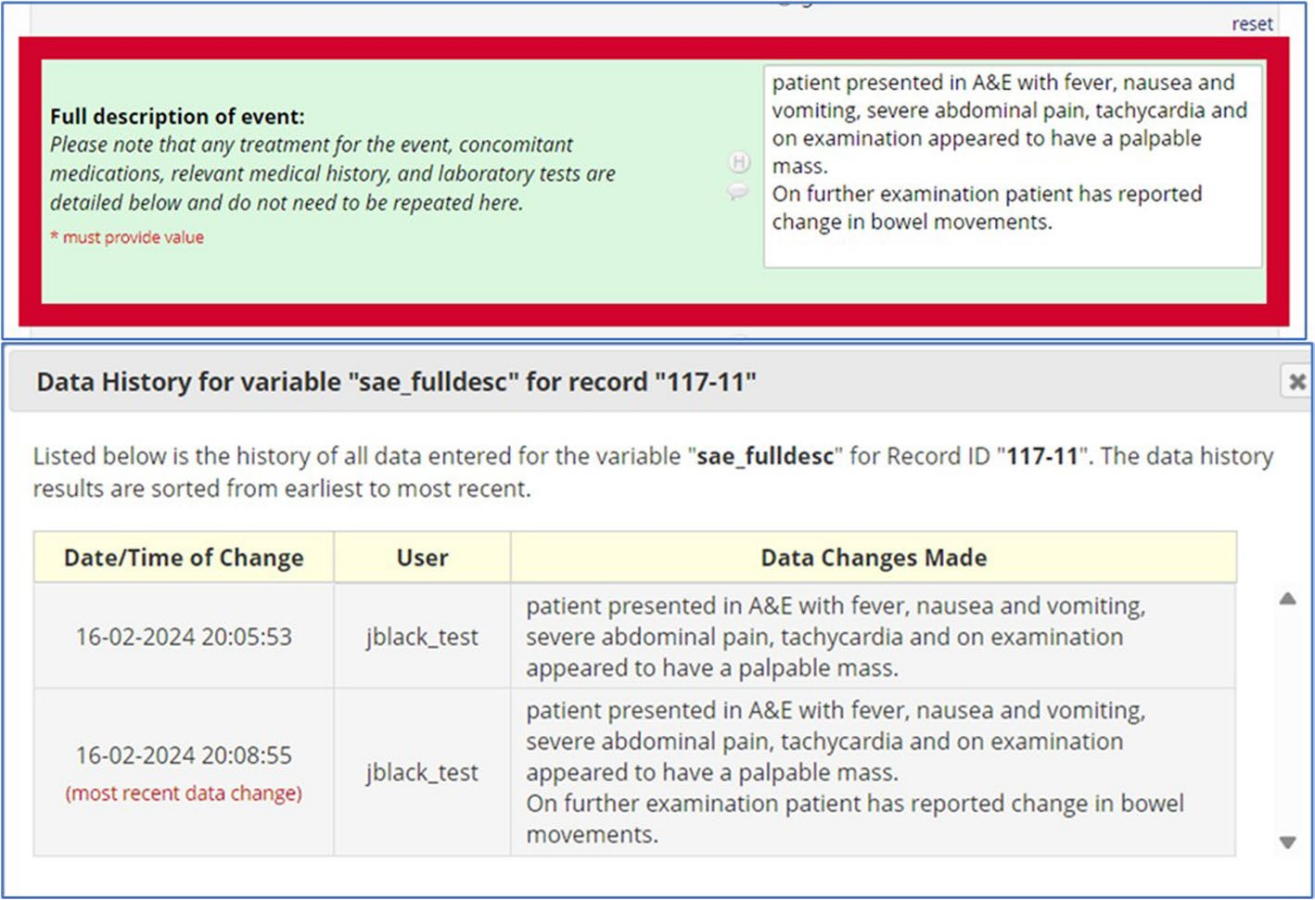
A highlighted box in red indicates that there has been changes in the field for full description of the event. The data history is revealed on clicking the H button next to the field

In Fig. 3, the full description of event is also surrounded by a red highlighted box, indicating that information has changed in this field. On clicking the ‘H’ button (history) next to the field, a history table is revealed shown what the data changes were, with an instant comparison of the updated information to the previous data in that same field.

**Fig. 4:**
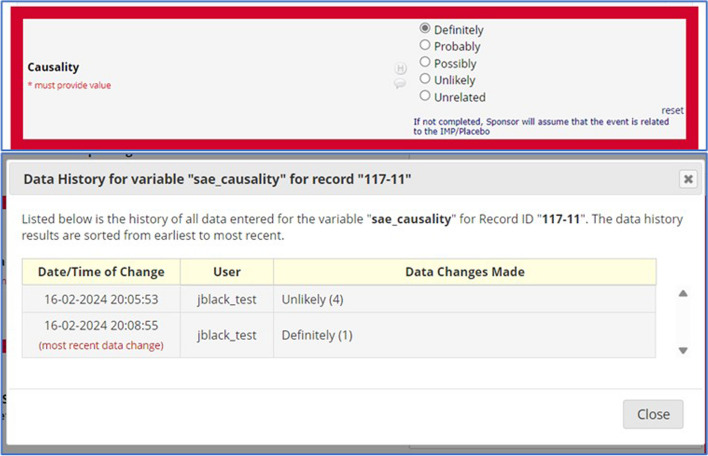
A highlighted box in red indicates that there has been a change in the assessment of causality. The data history is revealed on clicking the H button next to the field, revealing the previous entry

The information with regard to causality in Fig. 4 also appears to have a red highlighted box, indicating to the sponsor that the investigator has changed their assessment of causality to now stating ‘definitely’. On clicking on the ‘H’ button, a window, shown in Figure 7, reveals that causality has changed from unlikely to definitely, and hence this will alert the sponsor to review the SAE quicker, as a causality marked as definitely related means that the SAE has been elevated to a SAR, which means there needs to be an assessment of expectedness performed to establish whether the SAR is also a SUSAR, in which case this will need to be reported within the regulatory timeframe for reporting.

## Discussion

The aim of safety reporting in clinical trials is to identify, without delay and any ambiguity, any reactions in relation to the intervention that are serious and unexpected and that have the potential to jeopardise the safety of the participants and the trial as a whole. There is a huge drive to have safety reporting systems which are easy to use, easy to interpret and easy to process the data such that there is an instant recognition of which data is the final data set especially after any data has been changed or added to in the reporting tool. We believe that our solutions through the eSAE Project, where AE data populates the SAE system negating the need for double entry of information and where data changes are instantly identified by a colour code change, make it a project that streamlines the processing and reporting of accurate safety data. The eSAE Project working group identified several issues in the SAE pathway that needed to be addressed.

Inefficiencies in raising eSAEs from eAE forms: if an AE is considered to qualify as a SAE an eSAE form is required in addition to the initial eAE form. As these relate to the same event, much of the data will be the same, and so manual replication is (a) unnecessarily time-consuming and (b) at risk of transcription errors. The working group felt this process should be automated to be instant and accurate. This also has the added advantage that at the end of the trial there is no need for reconciliation between AEs entered in the study database and SAEs reported in the safety database, as both forms sit on the same platform and the SAE receives its initial data as an automatic pull from the AE form. SAEs are deemed finally closed when any queries raised by the CTU (sponsor) are answered by the site, the SAE has been marked by the site as ‘resolved’ or ‘recovered with sequelae’ and there is sign off from the site medically qualified doctor listed on the delegation log.

Difficulties linking related eAE and eSAE forms: if an AE does qualify as a SAE, the two forms must be clearly linked to prevent double-reporting. Manually created log numbers are at risk of being created out of sequence (particularly if it is a site responsibility to create the log number, and the site cannot see the log numbers used in other sites), so the working group was keen to automate this process.

Clear demarcation of data entry responsibilities: eSAE forms are split into sections to be completed by staff in several different roles (site staff, the central trial team, chief investigator/nominated person), and it is essential that edit access to each section is restricted to the appropriate user role (e.g. the CTU should not be able to edit data in the site-entered section). The working group felt it was important to configure the eSAE form at the field level to remove any opportunities for role-based errors.

Delays in notifying the sponsor of a SAE occurring: timely reporting to the sponsor is of paramount importance, but can be subject to the capacity and availability of the site team. The working group established that the notification process should be automated to ensure instant alerting of the sponsor when an eSAE form is submitted.

Challenges for chief investigators in identifying and reviewing changes made to eSAE forms from initial reporting: eSAE data may need to be expanded upon or updated over the course of several days or weeks, and chief investigators must review any changes in a timely manner to determine whether the key assessments of causality or seriousness should be amended. This is difficult given that the eSAE form contains several data points (any of which could be altered). It was a priority for the working group for the CI to be notified when any data had been changed and to clearly identify and flag changed data to allow an efficient review process.

All of the above solutions are configurable to meet the varying requirements of different studies. This eSAE Project is now our standard way of processing and streamlining SAE reporting and has already been rolled out in several CTIMP and nonCTIMP studies. As part of site initiation visits, the sites are provided with a set of slides showing screen shots of the whole process of how reporting SAEs will work and the impact of changing any data once they submit the form. Feedback from all users has been positive and encouraging that our solutions to eSAE reporting are resulting in a quicker and more streamlined way of processing data.

## Conclusion

We have developed a unique project within REDCap that allows for a quick, easy and efficient way to remotely report and review SAEs. The system developed negates the need for to re-entry of AE data for those AEs that also classify as an SAE, notifies the sponsor immediately as soon as an SAE is entered and/or changed in the system, and clearly identifies any changes/updates made. The eSAE Project has enabled streamlining and facilitation of the review of the SAEs in a timely manner. Our eSAE Project allows multiple users to interact with the SAE form reflecting their role and permissions whether they have data entry roles that of being the principal investigator, the clinical trials unit reviewer or a sponsor representative. This system has an added advantage that at the end of a trial there is no need to reconcile the SAEs with corresponding AEs as this is automated by the system in use, thus reducing any errors and any duplication of AEs and SAEs if two systems were in use for AEs and SAE reporting.

## Data Availability

Please contact the authors for further information.

## Abbreviations

AE: Adverse event
CTIMP: Clinical trial of an investigational medicinal product
CTU: Clinical trials unit
EDC: Electronic data collection
EU: European Union
MHRA: Medicines and Healthcare products Regulatory Agency
OCTRU: Oxford Clinical Trials Research Unit
REDCap: Research Electronic Data Capture
SAE: Serious adverse event
SAR: Serious adverse reaction
SUSAR: Suspected unexpected serious adverse reaction
UKCRC: United Kingdom Clinical Research Collaboration
